# Metabolic syndrome risk score and time expended in moderate to vigorous physical activity in adolescents

**DOI:** 10.1186/1471-2431-14-42

**Published:** 2014-02-14

**Authors:** Antonio Stabelini Neto, Wagner de Campos, Géssika Castilho dos Santos, Oldemar Mazzardo Junior

**Affiliations:** 1 Center for Health Sciences, Universidade Estadual do Norte do Paraná, Alameda Padre Magno, 841, Jacarezinho, Paraná 86.400-000, Brazil; 2 Department of Physical Education, Universidade Federal do Paraná, Curitiba, Paraná, Brazil

**Keywords:** Chronic Diseases, Lifestyle, Metabolic Syndrome, Students

## Abstract

**Background:**

The clustering of metabolic syndrome risk factors is inversely related to the amount of physical activity. However, the question remains as to how much daily physical activity is enough to prevent the onset of metabolic disorders in adolescents? Therefore, the objectives of this study were to associate the metabolic risk score with the moderate to vigorous physical activity (MVPA) and to identify the amount of daily physical activity to prevent the onset of the metabolic risk factors in Brazilian adolescents.

**Methods:**

The study involved 391 participants aged 10 to 18 years. Physical activity was measured by accelerometry. The counts obtained in the different activities were transformed into metabolic equivalents and classified as light (≥ 1.5 but < 3.0 METs), moderate (≥ 3.0 but < 6.0 METs) and vigorous (≥ 6.0 METs) activities. The continuous risk score for metabolic syndrome was calculated using the following risk factors: waist circumference, blood pressure, blood glucose, HDL-C and triglycerides.

**Results:**

Time spent in MVPA was inversely associated with the continuous risk score for metabolic syndrome (p < 0.05). Analysis of the ROC curve suggests that these adolescents must perform at least 88 minutes per day of MVPA.

**Conclusions:**

These findings reinforce previous evidence that physical activity relates to metabolic syndrome in adolescents. This population should be encouraged to gradually replace part of their sedentary time with physical activities.

## Background

Metabolic syndrome (MetSynd) is a set of simultaneous pathophysiological changes that increase the risk of chronic diseases [1] and is associated with increased risk of cardiovascular disease [2] and diabetes mellitus [3]. MetSynd can occur early in life; however, no conclusive evidence has indicated the causal factors in the pediatric population. Its main cause is not genetic but falls under modifiable risk factors, such as environmental and behavioral elements [4,5].

Thus, the World Health Organization has recently launched the Global Recommendations on Physical Activity for Health [6], which recommends that children and adolescents engage in moderate to vigorous physical activity (MVPA) for at least 60 minutes daily. Based on the hypothesis that greater amounts of physical activity are associated with better metabolic health indicators, some researchers assume that the maintenance of high levels of physical activity from childhood to adulthood allows for the maintenance of a healthy risk profile with lower rates of morbidity and mortality from cardiovascular disease and diabetes later in life [7-9].

However, the difficulty in defining the exact relationship between physical activity and MetSynd is due to factors, including a) difficulty in accurately measuring physical activity, as most studies have used recall questionnaires or self-administered diaries; b) lack of consensus in the literature regarding the criteria for diagnosis of MetSynd in children and adolescents; and c) lack of sensitivity of the cut-off points for defining individuals at risk for a particular condition.

In recent years, researchers have chosen to analyze the association between physical activity and MetSynd and its components using continuous rather than categorical data [10-13]. The adoption of the metabolic risk score seems to be plausible because it is statistically more sensitive and less susceptible to errors than dichotomous approaches [14].

Previous research reported that metabolic risk score were inversely associated with the total physical activity and its sub-dimensions of intensities [15-17]. However, the question remains as to how much daily physical activity is enough to prevent the onset of metabolic disorders in adolescents? Is the currently recommendation of 60 minutes per day of MVPA sufficient?

Overall, the objectives of this study were: a) to associate the MetSynd risk score with the continuous time spent engaging in MVPA assessed by accelerometry, and b) to identify the amount of daily physical activity to prevent the onset of metabolic risk factors in Brazilian adolescents. We hypothesize that increase in MVPA is associated with low MetSynd risk score, and that 60 minutes of physical activity per day is not sufficient to inhibit the onset of metabolic risk factors in adolescents.

## Methods

### Sample

The clustered, random sample was comprised of adolescents from both sexes, aged between 10 and 18 years (13.3 ± 1.7 years), who were enrolled in public and private schools in the city of Jacarezinho, PR. According to information provided by the Regional Education Center, in the 2010 academic year, there were 5,242 students enrolled in the elementary and high schools.

From the list provided by each school of the number of rooms, for the 5th to 8th grades and first to third years of high school, two classes of each year of education were randomly selected to participate. Before the assessments, the parents or legal guardians of the adolescents who agreed to participate completed and signed an informed consent authorizing the use of their data.

Subjects who had a family history of disease (i.e., biological parents or grandparents with diabetes, recognized cardiovascular disease, heart attack or sudden death) were excluded. By the end of the data collection, there were 391 adolescents with valid information. This study was approved by the Ethics Committee in Human Research of the State University of Maringa (UEM), number 668/2010, which is in accordance with the Declaration of Helsinki and Resolution 196/96.

### Instruments and procedures

#### Physical Activity

The physical activities were measured using an Actigraph accelerometer (GT3X, Pensacola, Florida, USA). Accelerometers were programmed to record the information at 60-second intervals. The participants wore the equipment on the hip at the height of the anterior iliac spine for 7 consecutive days. The values of counts/minute equal to zero for 30 minutes or more were excluded from the analysis on the assumption that the device was not being used [18]. The subjects who obtained no less than four full days of data, i.e., ≥ 600 minutes/day, with at least one valid weekend day, were included. The accelerometer is a valid and reliable instrument for measuring physical activity in adolescents in both the laboratory and during outdoor activities [19-21].

The counts obtained in the different activities were converted into metabolic equivalents (METs) using the equation developed and validated by Freedson and colleagues [22]. The cutoff points adopted for the intensities of physical activity were as follows: light (≥ 1.5 but < 3.0 METs), moderate (≥ 3.0 but < 6.0 METs) and vigorous (≥ 6.0 METs).

#### Anthropometric measurements

Participant height was assessed with a portable WCS stadiometer to the nearest 0.1 cm, and body mass was measured with a digital scale to within 0.1 kg. Waist circumference was measured at the midpoint between the last rib and the iliac crest [23].

#### Blood Pressure

Blood pressure was measured by the auscultatory method following the parameters established in the literature [24]. Systolic blood pressure (SBP) and diastolic (DBP) were measured in the subject's right arm. SBP and DBP were defined as the first and fifth phases of the Korotkoff sounds, respectively. The measurement was performed after the individual sat at rest for a period of 5 minutes, with the back supported, feet on the ground, and the right forearm supported with the cubital fossa at heart-level. Two readings were taken with an interval of 10 minutes between measurements, and the mean value between the two measurements was recorded.

#### Blood tests

A minimum of 10 hours fasting was required to participate in the tests. Blood samples were taken by venipuncture, processed and analyzed on the day of collection by the automated colorimetric enzyme method, using a COBAS MIRA Plus – ROCHE apparatus. The kits used for glucose and triglycerides were from “WIENER”, triglycerides TG Color GPO/PAP AA and AA Glucose enzyme. "Ebram" Quimicol - Ultra-Sensive kits were used to assess HDL-C.

#### Continuous risk score for MetSynd

For each risk factor, a Z score was calculated (individual value - sample mean/standard deviation of the sample). For the blood pressure, we used the average of SBP and DBP for calculating the score. Unlike the other components, a low value is unfavorable for HDL-C; thus, the calculation of the score was reversed (sample mean - individual value/standard deviation of the sample). The sum of the Z scores represents the score of continuous risk for Mets MetSynd (total score = waist Z score + BP Z score + glucose Z score + HDL-C Z score + triglycerides Z score). A lower risk score is indicative of a better metabolic profile.

### Statistical procedures

Data were analyzed using SPSS software version 15.0 for Windows, with the significance level set at p < 0.05 for all analyses. We used the Student t-test for independent samples to compare the rates of physical activity between the sexes. Kolmogorov Smirnov analyses verified the normality of the data set. Pearson correlation coefficients were calculated to assess the relationships between physical activity scores and continuous risk for MetSynd. Single-factor analysis of variance (ANOVA) compared the metabolic risk scores between quartiles of MVPA. Finally, ROC curves were used to determine the cutoff points in minutes/day of MVPA necessary to prevent the MetSynd (state variable risk score > 0).

## Results

Information on the characteristics of the sample is presented in Table 1. The boys were more physically active than the girls, according to time spent in physical activity of moderate intensity, vigorous intensity, and counts/minute. There were no significant differences between genders in recorded daily time and light activity. Regarding the nutritional status, 16.5% of them were overweight (male: 14.9%, female: 17.5%) and 9.3% were obese (males: 7%, female: 10.7%).

**Table 1:** Characteristics of participants and time spent in physical activity at different intensities

	**Male**	**Female**
	Mean ± SD	Mean ± SD
Height (cm)	158.3 ± 11.2	155.8 ± 8.1
Body Weight (kg)	50.0 ± 12.6	50.6 ± 12.3
Recorded Time (min/day)	827.0 ± 167.2	873.4 ± 166.1
Light Activity (min/day)	285.9 ± 69.0	281.7 ± 65.3
Moderate Activity (min/day)	96.1 ± 39.6	73.7^†^ ± 37.7
Vigorous Activity (min/day)	9.7 ± 8.8	6.1^†^ ± 6.8
Physical Activity (counts/min)	476.15 ± 174.0	373.32^†^ ± 152.2

^†^p < 0.05 between genders by Student t-test for independent samples.

The mean values for each risk factor of MetSynd separated by gender are shown in Table 2. There were no significant differences between the sexes. Considering the reference values suggested in the literature for adolescents, 14.1% had values of triglycerides ≥ 110 mg/dL (male: 14.9%, female: 13.6%), 22.3% showed HDL-C levels ≤ 40 mg/dL (male: 25.4%, female: 20.3%), 2.1% presented blood glucose ≥ 110 mg/dL (male: 3.5%, female: 1.1%), 23.4% showed values of systolic and/or diastolic blood pressure ≥ 90th percentile (male 22.8%, female: 23.7%), and 4.8% had the waist circumference ≥ 90th percentile (male 5.1%, female: 4.4%). From the entire sample, 33% presented at least one risk factor, 13.4% two risk factors, 2.4% three risk factors, and 1% four risk factors. The mean for metabolic risk scores for males and females are shown in Table 3.

**Table 2:** Risk factors for metabolic syndrome by sex

	**Male**	**Female**
	Mean ± SD	Mean ± SD
Waist Circumference (cm)	68.0 ± 8.1	66.8 ± 9.1
SBP (mmHg)	106.1 ± 12.9	104.5 ± 13.1
DBP (mmHg)	67.5 ± 14.4	67.6 ± 15.9
Glucose (mg/dL)	79.6 ± 9.0	78.2 ± 9.6
HDL-C (mg/dL)	49.0 ± 11.3	51.2 ± 11.5
Triglycerides (mg/dL)	74.2 ± 35.4	75.9 ± 35.7

SBP: Sistolic Blood Pressure; DBP: Diastolic Blood Pressure; HDL-C: High Density Lipoprotein Cholesterol.

No statistical differences between the genders by Student t-test for independent samples.

**Table 3:** Mean values of components of continuous metabolic risk score by sex

	**MALE**	**FEMALE**	**TOTAL**	
	Mean ± SD	Mean ± SD	Mean ± SD	
Z_Waist circumference	0.0099 ± 1.009	0.0048 ± 1.011	0.0068 ± 1.008	
Z_Blood Pressure	0.0249 ± 0.852	-0.0207 ± 0.978	-0.0029 ± 0.929	
Z_Glucose	0.0089 ± 1.007	0.0103 ± 1.001	-0.0098 ± 1.001	
Z_HDL-C	-0.0082 ± 1.005	-0.0014 ± 1.003	-0.0041 ± 1.002	
Z_Triglycerides	0.0009 ± 1.001	0.0025 ± 1.002	0.0019 ± 1.000	
**Z_TOTAL**	-0.0132 ± 2.849	-0.0047 ± 2.594	-0.0080 ± 2.692	

Z: standardized components of a continuous metabolic syndrome risk score.

HDL-C: High Density Lipoprotein Cholesterol.

No statistical differences between the genders by Student t-test for independent samples.

Table 4 presents the correlation coefficients between physical activity and continuous risk score of each component and the total risk score. A inverse association was observed between the practice of MVPA and the total risk score, indicating that the more time spent engaged in MVPA, the lower the continuous risk score.

**Table 4:** Correlation coefficients between the time expended in the physical activity sub-dimensions of intensities and standardized components of a continuous metabolic syndrome risk score

	**Z_WC**^**1**^	**Z_BP**^**2**^	**Z_GLU**^**3**^	**Z_HDL-C**^**4**^	**Z_TG**^**5**^	**Z_Total**
Light	-0.076	-0.149*	0.042	-0.056	-0.018	-0.093
Moderate	-0.121	-0.162*	-0.116	-0.071	-0.046	-0.191**
Vigorous	-0.175**	-0.115	-0.029	-0.064	0.040	-0.124
Moderate-Vigorous	-0.139*	-0.166*	-0.109	-0.075	-0.034	-0.193**

^1^Standardized Waist Circumference; ^2^Standardized Blood Pressure; ^3^Standardized Glucose; ^4^Standardized High Density Lipropotein Cholesterol; ^5^Standardized Triglycerides.

*p < 0.05; **p < 0.01; Pearson correlation coefficients.

**Figure 1:**
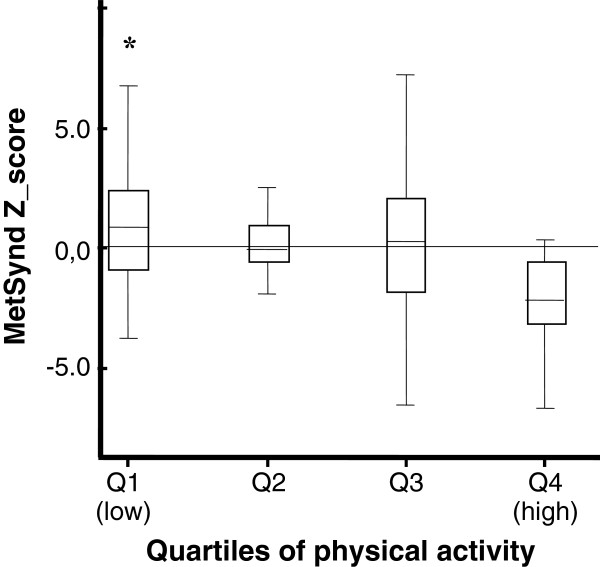
**Continuous risk score for metabolic syndrome according to quartiles of moderate to vigorous physical activity for boys (Mean and SD).** *Significant difference for the fourth quartile (more active) at p < 0.01. Single-factor analysis of variance (ANOVA).

**Figure 2:**
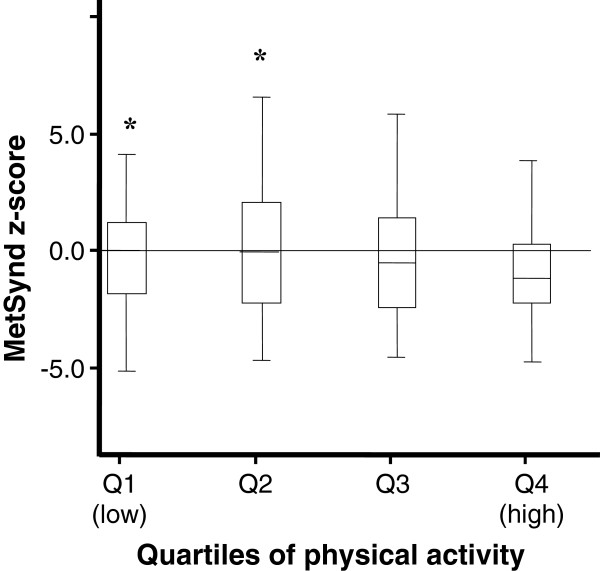
**Continuous risk score for metabolic syndrome according to quartiles of moderate to vigorous physical activity for girls (Mean and SD).** *Significant difference for the fourth quartile (more active) at p < 0.01. Single-factor analysis of variance (ANOVA).

For comparison of the values of continuous risk score between the levels of physical activity, the adolescents were divided into quartiles according to MVPA. The Figures 1 and 2 indicate that in both sexes, young people belonging to the fourth quartile of physical activity (more active) had lower mean values of the risk score than their peers belonging to the first quartile (less active) (boys: F = 5.67, p < 0.01, girls: F = 3.80, p < 0.01).

**Figure 3:**
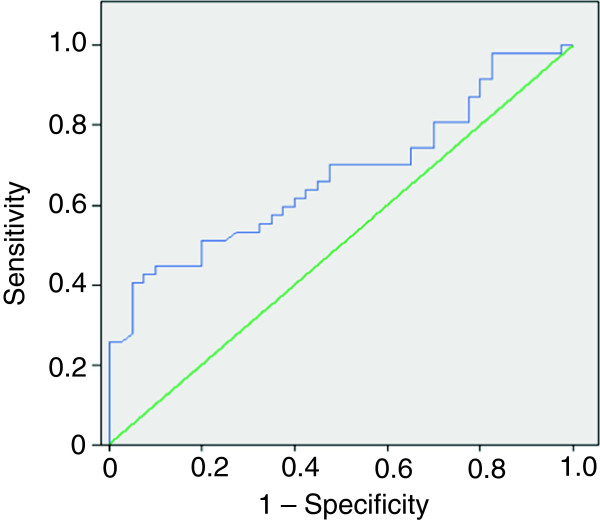
ROC Curve between MVPA versus MetSynd z-score.

The amount of physical activity determined by analysis of the ROC curve was that adolescents must perform at least 88 minutes per day of MVPA to maintain a lifestyle that promotes a healthy metabolic profile. Specificity and sensitivity are depicted in Figure 3.

## Discussion

In the present study, the time spent in MVPA per day was inversely associated with the continuous total risk score. This finding has been previously demonstrated [10,15-17], indicating that individuals who are more physically active present lower total metabolic risk scores. In addition, when we separated the subjects by quartiles of MVPA in both sexes, the students belonging to the fourth quartile (more active) demonstrated lower mean scores than their peers in the first quartile (less active). The least active group had twice the chance of diagnosis of MetSynd compared to the most active peers (data not shown).

There are two hypotheses attempting to explain the possible causal relationship between physical activity and health in children and adolescents [25]. First, children with low levels of physical activity are more likely to develop degenerative diseases in adulthood. Thus, the practice of physical activity during childhood can induce biomechanical, physiological and psychological changes, which manifest themselves as chronic beneficial adaptations that persist throughout adulthood. Second, the habit of physical activity acquired during childhood persists into adulthood and plays a vital role in the prevention of cardiovascular disease.

This question was raised over two decades ago, but to date, the minimum amount of physical activity required to prevent and treat the clustering of metabolic risk factors in the pediatric population remains uncertain. Since 2000, the U.S. Department of Agriculture has recommended that children and adolescents should participate in at least 60 minutes of MVPA on most days of the week, preferably daily [26]. This recommendation was endorsed by the Global Recommendations on Physical Activity for Health, 2010 [6]. However, as this amount of physical activity is easily achieved by most adolescents, especially younger children, the question remains: Is 60 minutes per day of MVPA sufficient to provide a healthy metabolic profile?

Andersen et al. [11] conducted a survey with 1732 schoolchildren to evaluate the association of objectively measured physical activity with the aggregation of risk factors for cardiovascular disease. The authors found a progressive increase in the values of the odds ratio for the clustering of risk factors compared with the most active quintile (5th quintile: 131 minutes per day; 4th quintile: 88 min.; 3rd quintile: 70 min., 2nd quintile: 53 min.; 1st quintile: 34 min.). The amount of physical activity necessary to prevent the clustering of risk factors for cardiovascular disease in adolescents should be 90 minutes of daily physical activity of at least moderate intensity rather than the current recommendation of one hour per day. This recommendation is supported by the findings of the present study in which we observed that adolescents must perform at least 88 min / day of MVPA to maintain a lifestyle that promotes a healthy metabolic profile.

Based on studies assessing the association between PA and metabolic risk, it seems logical that young people should be encouraged to replace some sedentary time with light physical activity and then proceed to moderate-intensity activities. A gradual increase in PA must achieve a sufficient level to normalize the metabolic profile, using unstructured and enjoyable activities to maintain exercise adherence [14,27].

A limitation of this study was the employment of the sample mean value in the calculation of the MetSynd risk score; thus, caution is needed in extrapolating results. Key strengths of this study were the representative sample of Brazilian adolescents and the use of an objective measure of physical activity.

Future research in this area should investigate the optimal amount of physical activity to promote health in children and adolescents. Alternative designs are preferred because cross-sectional studies do not guarantee the temporal precedence of variables and limit the extrapolation of the observations.

## Conclusions

The results of this study reinforce previous evidence that physical activity, especially activity of moderate to vigorous intensity, is inversely related to the continuous risk score of MetSynd in adolescents. Based on the analyses conducted, it is suggested that adolescents perform at least 88 min/day of MVPA to promote a healthy metabolic profile. These activities should include games, sports, recreation, planned exercises, and transportation, both in the context of school and in the community.

## Competing interests

The authors declare that there are no conflicts of interests.

## Authors’ contributions

Dr. ASN: responsible for the collection, analysis, and interpretation of data, as well as for drafting the manuscript; Dr. WdeC: analysis and interpretation of data and also in the critical revision of the manuscript; GCdosS: contributed to the data collection and in the writing of the manuscript. Dr. OM revised the manuscript for intellectual content and contributed to the writing of the manuscript. All authors read and approved the final manuscript.

## Pre-publication history

The pre-publication history for this paper can be accessed here:

http://www.biomedcentral.com/1471-2431/14/42/prepub
